# Ectromelia Virus Infections of Mice as a Model to Support the Licensure of Anti-Orthopoxvirus Therapeutics

**DOI:** 10.3390/v2091918

**Published:** 2010-09-03

**Authors:** Scott Parker, Akbar M. Siddiqui, George Painter, Jill Schriewer, R. Mark Buller

**Affiliations:** 1 Department of Molecular Microbiology and Immunology, Saint Louis University School of Medicine, 1100 S. Grand Blvd., St. Louis, MO, 63104, USA; E-Mails: scott9379@gmail.com (S.P.); amsiddiq@gmail.com (A.M.S.); jill.schriewer@gmail.com (J.S.); 2 Chimerix Inc., 2505 Meridian Park Way, Suite 340, Durham, NC, 27713, USA; E-Mail: gpainter@chimerix-inc.com

**Keywords:** ectromelia, variola, monkeypox, animal model, mousepox, infection route, antiviral, CMX001, ST-246

## Abstract

The absence of herd immunity to orthopoxviruses and the concern that variola or monkeypox viruses could be used for bioterroristic activities has stimulated the development of therapeutics and safer prophylactics. One major limitation in this process is the lack of accessible human orthopoxvirus infections for clinical efficacy trials; however, drug licensure can be based on orthopoxvirus animal challenge models as described in the “Animal Efficacy Rule”. One such challenge model uses ectromelia virus, an orthopoxvirus, whose natural host is the mouse and is the etiological agent of mousepox. The genetic similarity of ectromelia virus to variola and monkeypox viruses, the common features of the resulting disease, and the convenience of the mouse as a laboratory animal underscores its utility in the study of orthopoxvirus pathogenesis and in the development of therapeutics and prophylactics. In this review we outline how mousepox has been used as a model for smallpox. We also discuss mousepox in the context of mouse strain, route of infection, infectious dose, disease progression, and recovery from infection.

## 1. Ectromelia virus

Infectious ectromelia (ECTV) was identified in 1930 when the mouse was first introduced as an experimental laboratory animal [1]. Wild populations of rodents in Europe are suspected to be infected naturally with ECTV and the virus is transmitted easily among wild and laboratory populations under experimental conditions [2]. Mice that survive the acute phase of disease develop an exanthematous rash called mousepox that is similar to that of smallpox. ECTV causes an acute epizootic disease in mouse colonies in Europe, Japan, China and the US [3–5]. Laboratory studies have shown that ECTV, like Variola virus (VARV) infections in humans, has a very narrow host range, infecting only certain mouse species [6]. The genetic similarities of VARV and ECTV, and the commonality of disease course led to ECTV being proposed as a model of smallpox and exanthematous diseases in the 1940s. In this capacity, mousepox provides an excellent model for testing anti-orthopoxvirus therapeutics and prophylactics [7–8]. This rudimentary understanding of ECTV infection of the mouse and spread to internal organs during the disease incubation period still forms the conceptual basis for the incubation period of smallpox and human monkeypox. Studies from a succession of investigators in the last five decades have resulted in a detailed description of the virologic and pathologic disease course in genetically susceptible (A, BALB/c, DBA/2, and C3H/He) and resistant (C57BL/6, SKH1 and AKR) inbred and outbred mice; identification and characterization of important cell-mediated and innate responses for recovery from infection [9–19]; and the discovery of rmp-1, rmp-2, rmp-3 and rmp-4 loci which govern resistance to severe mousepox [20–23]. Varying mouse genotypes, virus strain and dose of virus result in distinct disease patterns for a given route of infection.

### Mousepox as a model of smallpox

**Figure 1.**
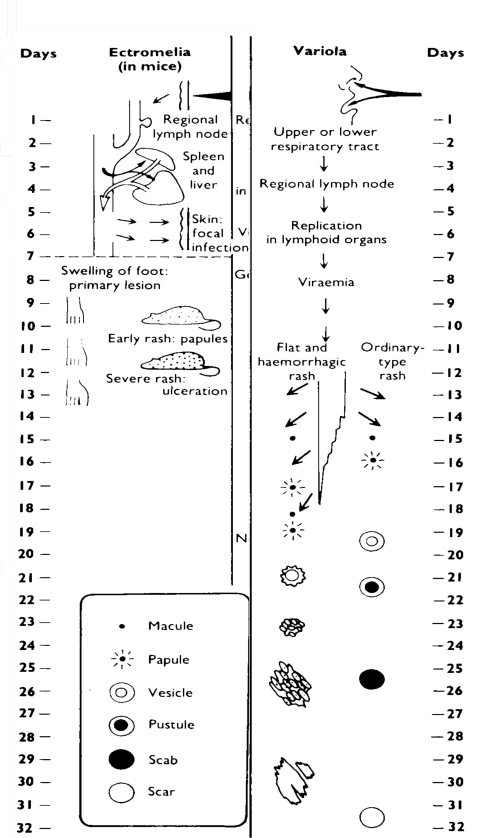
The spread of virus around the body and the evolution and healing of skin lesions in the mousepox system and in smallpox in humans (courtesy of the WHO [24]).

Mousepox has at least four features similar to smallpox (Figure 1). First, a relatively small dose of virus is required to initiate disease in the upper and lower respiratory tract (although the actual dose required to initiate smallpox is unknown, it is generally accepted to be a low dose [24]). Second, following a low dose intranasal (IN) infection there is no obvious lung involvement during the course of early disease (data not shown). Third, virus can be detected in respiratory gases during the pre-exanthem period [25]. And fourth, both diseases present with a characteristic exanthematous rash, although in the case of mousepox, rash development is dependent on a number of parameters including mouse strain, virus strain, route of inoculation, and virus dose [3]. Mousepox differs from smallpox in at least two features following respiratory tract infection. First, the disease course in mousepox is shorter as compared to smallpox. Death in fatal cases of mousepox usually occur 7 to 14 days p.i., whereas deaths in ordinary smallpox occur approximately 18 to 22 days p.i. [24]. Second, the major lesions in mousepox are observed in the liver and spleen, whereas these organs are relatively uninvolved in smallpox [4,24].

## 2. The animal efficacy rule

Naturally occurring smallpox was eradicated in the late 1970s by a global vaccination program sponsored by the WHO. Human monkeypox, although on the rise, is still sporadic and usually occurs in the tropical rain-forests of Africa [26]. Therefore, there are insufficient numbers of accessible human orthopoxvirus infections for clinical efficacy trials. In recognition of this problem, the Food and Drug Administration (FDA) promulgated the so-called “Animal Efficacy Rule”, which acknowledges that therapeutics and prophylactics against NIAID (National Institute of Allergy and Infectious Diseases) Category A biothreat agents cannot be licensed under the usual regulatory standards (21 CFR 314 or 601). (United States Code of Federal Regulations title 21, part 314, subpart I, Federal Register, 2002). The Animal Efficacy Rule permits the use of well-controlled animal efficacy data to support an application for licensure of drugs and biological products intended to treat, or prevent, serious or life-threatening conditions in humans resulting from exposure to biological, chemical, radiological or nuclear substances. Product Licensure requires that the Animal Efficacy Rule be utilized if human challenge or protection efficacy trials to test the product would be unethical due to the risks associated with exposure, or when clinical field trials are unfeasible (e.g. VARV no longer circulates in human populations). Although the selection of animal models is left up to the scientific judgment of the principal investigator, a typical choice would involve at least one rodent and non-human primate model.

The Animal Efficacy Rule presents regulatory hurdles for licensure of vaccines and poxvirus antivirals [27–28]. The criteria for animal data use in licensure of products under the Animal Efficacy Rule is stated in Table 1, and is matched to the realities of smallpox product development in animal models [28]. The first three sections of Table 1 apply equally to vaccines and antivirals with the fourth section specifically addressing issues relevant with antivirals. Although the available animal models can be characterized in great detail using modern molecular and immunologic techniques, little is known about the molecular and cellular basis of the pathogenesis of VARV or monkeypox virus (MPXV), especially during the 10–12 day incubation period that was modeled on a 1950s understanding of mousepox. There is no single animal model that mimics smallpox and human monkeypox accurately. The animal models differ from human disease in the infectious dose required to initiate infection, tissues targeted for pathology, and duration of disease. The licensure of smallpox antivirals requires a profound and sustained research effort, and constant open dialog among the drug sponsor, regulatory authorities and government agencies to reduce the Animal Efficacy Rule to practice [28].

In the case of antiviral development, the sole use of animal efficacy data as a means of establishing an effective human dose is problematic [28]. Because there are no pharmacodynamic responses in animal models that can predict the human response to an anti-orthopoxvirus drug, human dose selection must be based on kinetics. For example, the hexadecyloxypropyl lipid side chain of CMX001 is subject to oxidative catabolism in mice, rats, rabbits, monkeys, and humans. However the extent of the catabolism, and the precise intermediates along the catabolic pathway isolated from plasma vary significantly between species. This results in very different plasma exposures in animals of different species given the same mg/kg dose. Consequently it is difficult, if not impossible, to calculate directly an efficacious dose in a given species based on the efficacious dose determined experimentally in another. However, it is possible to scale efficacious plasma exposure directly between species using the pharmacokinetic parameters of C_max_ and AUC_o⇒∞_. In mice, for example, an efficacious dose of CMX001 against an ECTV infection is 5 mg/kg. This dose results in a plasma exposure to CMX001 with a C_max_ of 29 ng/ml and an AUC_o⇒∞_ of 60 ng/ml/hr. In order to achieve this plasma exposure in humans requires a significantly lower dose of 1 mg/kg. This difference is largely due to differences in degree of oxidative catabolism between mice and humans. It is noteworthy that this dose in humans has shown a clinical efficacy against vaccinia virus (VACV), adenovirus and cytomegalovirus infections, supporting the idea that activity against ECTV in mice can be used to help establish an efficacious human dose against relevant orthopoxviruses as well as other double stranded DNA virus infections.

## 3. Mousepox severity is dependent on mouse strain

We and others have previously shown that a very low dose of ECTV virus (<10 Plaque forming units, PFU) delivered by the footpad (FP) and subcutaneous (SC) routes induces uniform mortality in A/Ncr mice by day 8 p.i. [29–30]. Conversely, infections of the C57BL/6 and SKH-1 strains via the SC/FP routes result in a milder, non-lethal illness with high LD_50_ (lethal dose 50%) values of >1 × 10^6^ PFU and >2000 PFU, respectively [31]. Interestingly, the response to a FP infection in the C57BL/6 strain is so strong that it can protect IN infected mice from lethal infections when administered at least 24 hours after the IN infection (data not shown and [29]).

The vastly different disease outcomes following FP/SC infections have been most thoroughly studied in the C57BL/6 and A/Ncr strains. Following a 100 PFU FP infection in the A/Ncr strain, infectious virus can be detected in the spleen by 2 days p.i., in the liver by 4 days p.i. and in the lungs by 6 days p.i.; however, in the C57BL/6 strain very low levels of infectious virus are detected in the liver at day 5 p.i. but none is detected in the spleen or lung. Conversely, when the A/Ncr and C57BL/6 strains are infected IN with 1000 PFU, 100% mortality is observed by day 8 p.i. and day 14 p.i. respectively. And, infectious virus can be detected from day 2 and day 4 p.i. in the liver, spleen and lung of A/Ncr and C57BL/6 mice, respectively; however, viral titers are consistently 1–2 logs lower in C57BL/6 mice compared to equivalent tissues from A/Ncr mice [29].

Some insights into the reason for the different disease outcomes following FP infections can be gleaned by examining the draining popliteal lymph nodes (PLN) of the C57BL/6 and A/Ncr strains. We found that there was minimal overlap in the host gene transcription pattern between the C57BL/6 and A/Ncr PLNs at 6, 12 and 24 hours p.i. (data not shown). Also, several cytokines, such as IFNγ and Rantes, were elevated in C57BL/6 PLN cells by 24–48 hours p.i. but were only slightly elevated or unchanged in A/Ncr PLN cells, respectively [29]. Furthermore, PLN cells from C57BL/6 mice present antigen to pre-primed CD3+ splenocytes by 24 hours p.i.; however, no such presentation could be detected in the equivalent experiment using A/Ncr mice up to 72 hours p.i. [29]. That said, the A/Ncr PLN was not completely unresponsive because IL-9 levels were elevated by 24 hours p.i. but remained unchanged in the PLN of the C57BL/6 strain [29]. These cytokine response differences could indicate a Th1 and Th2 biased response in the C57BL/6 and A/Ncr strains, respectively. Thus, following a FP infection the protective innate/adaptive immune response is initiated earlier, and is qualitatively different, in the C57BL/6 mouse strain as compared to the A/Ncr strain.

## 4. Importance of route and infectious dose in animal models of smallpox

### 4.1. Route of infection

The pathogenesis of an infectious agent is greatly affected by its route of infection. VARV causes a systemic, fulminant disease following a respiratory tract infection with a case-fatality rate of 10–30%. Epidemiologic studies suggested that this infection is mediated by large-droplet aerosol that would initiate infection in the upper respiratory tract, which is modelled by the IN route of infection. In contrast, infection through the skin results in a systemic infection, but with a milder disease course and case-fatality rate of <1% [24]. Therefore, it is important that the route of infection and the challenge virus used to evaluate antivirals recapitulate the pathogenesis of the natural disease and the host response to it. Certain rabbit/rabbitpox virus and monkey/monkeypox virus models utilize intradermal and intravenous (IV) routes of infection, respectively, that remove the seeding and early stages of viral replication in the respiratory tract [32–34].

The A/Ncr strain is sensitive to lethal ECTV infection by IV, IN, FP/SC and IP routes. The C57BL/6 mouse is resistant to FP/SC ECTV infections but is highly sensitive to IV (LD_50_ = 10,000) and IN (LD_50_ = 100 PFU) infections [31]. The IV route has also been evaluated in the ECTV model but has some distinct disadvantages compared to the natural route of VARV transmission. Primarily, the instantaneous viremia shortens the incubation and prodromal phases of the disease. Thus, the battery of ECTV encoded evasion molecules that dampen the innate response to infection in the skin are likely compromised or completely bypassed. It has also been shown that the virus inoculum is rapidly inactivated by complement, which thereby reduces the viral challenge dose and likely facilitates accelerated antigen presentation. In our hands, we observed six-fold increases in blood CD8+ T cells secreting IFN-γ following IV infections as compared to the IN route (data not shown). Moreover, one report reveals that following an IV challenge the liver clears 95% of ECTV from circulation within five minutes of injection. In the following hour, most of the viral antigen in the liver becomes undetectable by immunefluorescence and viral infectivity decreases by 90%. Such rapid removal further supports the hypothesis that the virus has been recognized and tagged for immune adherence and destruction [35–36].

**Figure 2.**
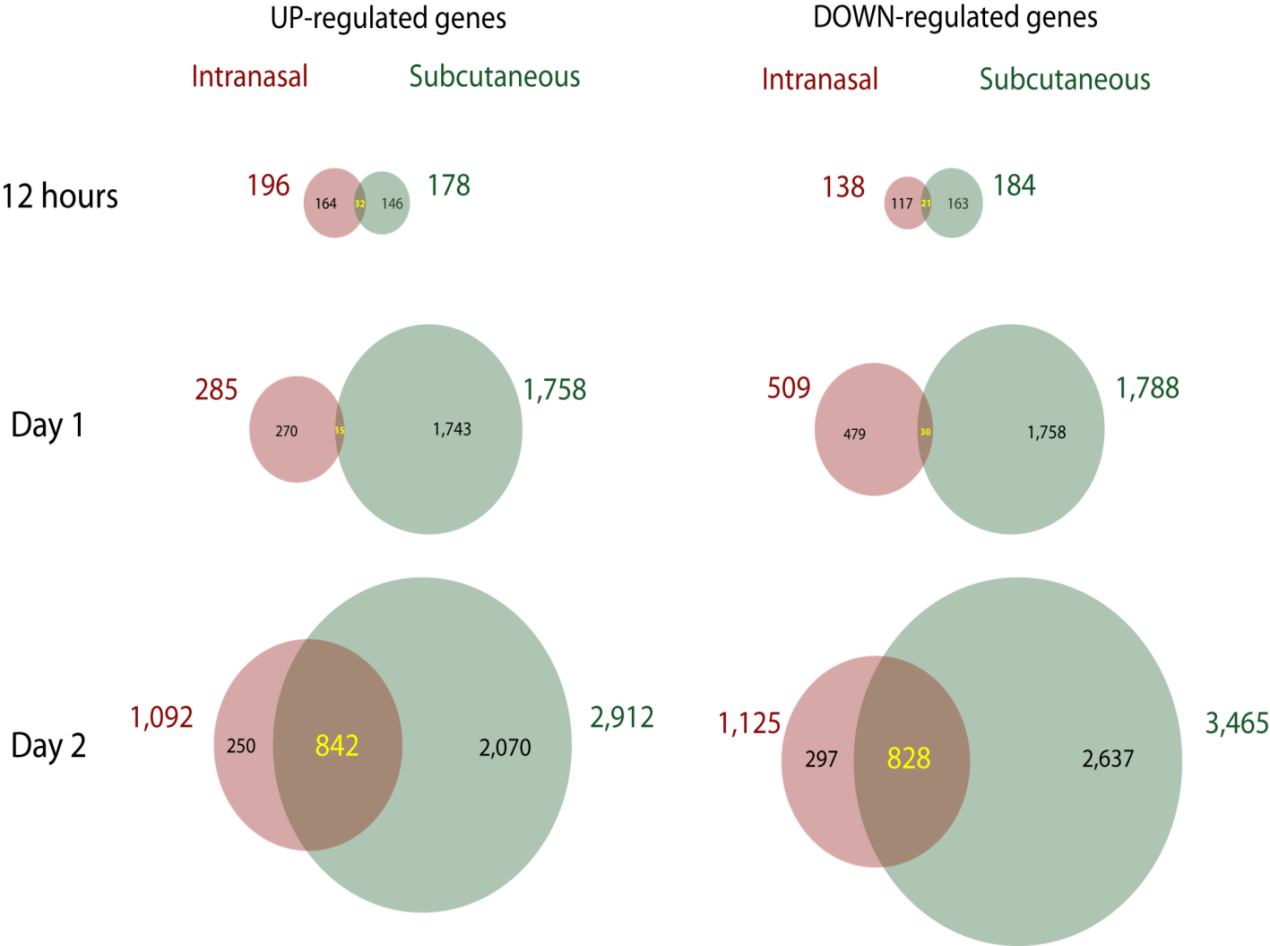
Gene chip arrays were used to measure up-regulated and down-regulated genes in the MLN of C57BL/6 mice infected IN or SC with ECTV at 12 hours, day 1 and day 2 p.i.

The importance of infectious route has been studied in the C57BL/6 mouse following an IN infection or a SC infection in the nose. Rapid changes are observed in the transcriptional pattern of cells isolated from the mandibular lymph node (MLN) which drains both sites of infection (Figure 2). Arrival of the virus at the MLN following a SC infection occurs at 12 hours p.i. as measured by infectivity titers and GFP+ cells, but is delayed until day 3–4 p.i. (measured by GFP+ cells) and day 1–2 p.i. (measured with titers) following IN inoculation (data not shown). As well as arriving at the MLN earlier, a SC infection results in the arrival of the virus at the spleen, liver, kidney and lung approximately 2 days earlier than it does following an IN infection; however, titers remain 1–3 logs lower in tissues from a SC infection compared to those from an IN infection. Furthermore, the cytokine profiles in the plasma and at the MLN are different for each route. For example, C57BL/6 mice infected via the SC route have detectable plasma IFNγ by day 1 p.i. which peaks at day 4 p.i. followed by a rapid reduction in levels by day 6 p.i. In contrast, following an IN infection, plasma IFNγ cannot be detected until day 3 p.i. but is followed by a rapid increase which continues until death. At the MLN, IFNγ, IL-6, KC (keratinocyte chemoattractant, CXCL1), and MCP-1 (monocyte chemoattractant protein-1, CCL2) are increased following a SC infection, but remain at control levels following an IN infection; Rantes (regulated upon activation normal T cell expressed and secreted, CCL5), however, is increased significantly at day 6 and day 7 in mice infected by the SC and IN routes [31]. The dramatic route-dependent differences in the host response to infection argues that animal models of smallpox should use the same infection route.

### 4.2. Infectious Dose

Based on several lines of evidence, the infectious dose of VARV is likely very low [26]. For example, infectivity was detected in oropharygeal secretions of infectious smallpox patients between two and nine days from onset of fever, and titers rarely were >10^5^ PFU/ml [24]. The virus was virtually undetectable in the air of smallpox wards unless measured within a short distance of the patients mouth [37–38]. Also, epidemiologic studies of the Aralesk, Meschede, and University of Birmingham smallpox outbreaks support a low infectious dose [24,39]. And finally, experimental studies with other orthopoxviruses have initiated infections with small doses of virus: 4 PFU of VACV (Westin Reserve strain) in rabbits, 15 PFU of rabbitpox (Utrecht Strain) in rabbits and 0.6 PFU of ECTV in mice [33,40]. Based on this data, animal models of smallpox should employ relative low challenge doses.

## 5. Indicators of disease progression and recovery from infection

### 5.1. Disease progression

Disease biomarkers provide a good method to monitor disease progression and the efficacy of antiviral therapies. Biomarkers have been most thoroughly studied in the A/Ncr and C57BL/6 mice following an IN infection and include: 1) AST (alanine aminotransferase) and ALT (aspartate aminotransferase) which give an indication of liver damage; 2) ECTV DNA can be detected and quantified with PCR (polymerase chain reaction) from whole blood as early as 4 days p.i.; and 3) Weight change provides a good trailing indicator of morbidity (see Table 2) [40]. Monitoring of core body temperature by telemetry has not provided a robust measure of disease progress work [31].

### 5.2. Recovery from infection

Recovery from IN infection is easily monitored by observing increases in animal body weight and by measuring: 1) blood neutrophilia; 2) serum IFN-γ; 3) ALT/AST; 4) infectivity titres; 5) DNA genome equivalents; and 6) levels of circulating IFN-γ secreting CD4 and CD8 T cells (Table 2). Importantly all of these measurements can be made from 100 μl of blood obtained from the submandibular vein without the need to sacrifice the animal [40]. To determine if the antiviral treatment has an effect on development of immunity to ECTV, we also monitor the antibody and cell-mediated memory responses at ∼60 days p.i., and the ability of the surviving antiviral-treated mice to resist a second ECTV challenge (∼1000x LD_50_).

## 6. Selecting a trigger for therapeutic intervention

Although various biomarkers have been evaluated to stage disease progression, the trigger for intervention has not been linked to an outwardly observable clinical sign of disease. The use of a “disease-defining manifestation” relevant to human disease to initiate therapy in an animal model is important for the generation of efficacy data under the Animal Rule as described in a 2009 FDA guidance document [41]. In an animal model of smallpox/human monkeypox the appearance of rash would be an ideal trigger as it appears 10–12 days following infection and contributes to clinical differential diagnosis. Observation of rash in C57BL/6 mice is difficult to visualise due to the presence of hair, although waxing the mice at various stages p.i. can been used to more easily see a rash-like pathology which presents itself from approximately day 7–14 p.i., depending on challenge dose. Another strain that has been used to evaluate antivirals is the hairless SKH1 strain. SKH1 mice respond to ECTV infection in a strikingly similar way to that of the C57BL/6 strain, *i.e.,* the mice are resistant to FP/SC infections but are sensitive to IN infections (LD_50_ = 100 PFU). Furthermore, the manifestation of the rash in the SKH1 strain is much more obvious and wide-spread compared to the rash-like pathology in the C57BL/6 strain which can present with as few as 10 lesions. Preliminary data indicate that rash appearance in these mouse strains occurs too late in disease to be used as a trigger for therapeutic intervention with an antiviral [31].

## 7. Evaluation of prophylactics and therapeutics in the ectromelia model

### 7.1. Efficacy testing in ECTV infected immunocompetent mice

The majority of recent efficacy studies have been performed in the A/Ncr, C56BL/6 and SKH1 strains. Although the A/Ncr strain’s sensitivity to SC infections, and its general hyper-sensitivity to ECTV, does not model VARV/MPXV infections in humans, it does provide a platform for the testing of drugs in mice infected with viral doses several thousand times higher than the LD_50_. We and others have used the A/Ncr strain to evaluate two promising orally bioavailable antivirals that have different antiviral modes of inhibiting orthopoxviruses; namely, ST-246 and CMX001 [42–43]. We found that following a 50 PFU IN infection, a daily 4 mg/kg dose of CMX001 initiated on the day of infection and continued for 5 days could protect all A/Ncr mice. With ST-246 we found that following a 3 PFU IN infection all A/Ncr mice were protected when dosed with a daily 100 mg/kg dose of ST-246 for 10 days commencing on the day of infection. A delayed CMX001 dosing regimen was therapeutically effective as late as 3 and 6 days p.i. in SKH1 and C57BL/6 mice, respectively, and ST-246 was therapeutically effective at 6 days p.i. in C57BL/6 mice.

### 7.2. Efficacy testing smallpox antivirals in immunodeficient animals

Immunocompromised patients infected with orthopoxviruses are a challenge to treat therapeutically as optimal antiviral efficacy is dependent on a functioning immune system. In certain immunocompromised patients, monotherapy evolves into a combination therapy due to the lack of clinical response. For example, a child with eczema vaccinatum was treated sequentially with VIG, cidofovir (CDV) and ST-246 prior to clinical progress and recovery [44]. Similarly, a case of progressive vaccinia, a rare and often fatal adverse event to vaccination was treated sequentially with VIG, ST-246, Imiquimod, and CMX001 prior to clinical progress and recovery [45]. These two clinical cases involving patients with varying degrees of immunodeficiency, and the available antiviral studies using immunodeficient animal hosts suggest that more research is needed to evaluate combination antiviral therapies against poxviruses [46–51]. This is particularly relevant as a significant portion of the population are immunocompromised [52].

Nude and SCID mice, and mice treated with ionizing radiation or cytostatic drugs, have been used to evaluate the efficacy of smallpox antivirals in immunodeficient hosts. Another approach is to use an ECTV recombinant expressing IL-4 (ECTV-IL-4), which induces a profound immunocompromised state in all tested mouse strains whether genetically resistant or previously vaccinated with a smallpox vaccine [53]. The evaluation of CMX001 and ST-246 in this model found that standard monotherapy failed to protect against lethal ECTV-IL-4 infections; however, the prophylactic combined administration of CDV or CMX001 and ST-246 significantly protected against lethal IN or FP challenges with ECTV-IL-4, as did combination therapy with CDV and an anti-IL-4 monoclonal antibody [31]. The advantage of this approach is that the host is an immunocompetent mouse strain that requires no ancillary treatments to induce immunosupression which is a consequence of global effects of virally produced IL-4 on the innate and adaptive immune system.

## Figures and Tables

**Table 1.** Elements of the animal efficacy rule^1^.

**Criteria for use of animal model**	**Issues relating to smallpox**
There is reasonably well understood pathophysiological mechanism for the toxicity of the substance and its prevention or substantial reduction by the product.	Scientific knowledge is limited as the last cases of endemic smallpox occurred in 1949 in the USA, and 1977 worldwide, prior to the age of molecular biology and immunology.
The effect is demonstrated in more than one animal species expected to react with a response predictive for humans, unless the effect is demonstrated in a single animal species that represents a sufficiently well characterized animal model for predicting human response.	VARV naturally infects only humans; experimental infection of nonhuman primates is forced. Animal models using related orthopoxviruses produce disease with similarities to smallpox, but the pathogenesis varies depending on the animal species, the characteristics of the infecting virus and the route of infection. No one animal model has been established that completely mimics human disease.
The animal study endpoint is related clearly to the desired benefit in humans, generally the enhancement of survival or prevention of major morbidity.	There are no animal models for the major morbidities of smallpox. Orthopoxvirus doses sufficient to produce 100% mortality in animal models shorten the incubation period substantially in most animal models, thus making it difficult to study the effect of post-exposure intervention. Interpretation of mortality studies in animals are limited by the ethical requirement to euthanize moribund animals.
The data or information on the pharmacokinetics and pharmacodynamics of the product or other relevant data or information, in animals and humans, allow selection of an effective human dose.	The specific pharmacodynamic response related to antipoxviral activity cannot be measured in uninfected humans for the purpose of selecting an effective dose. Pharmacokinetics in the animal species used in orthopoxvirus infection models may not be the most relevant for dose selection in humans.

1 Adapted from Future Virology (2006) 1(2) 173–179 with permission of Future Medicine Ltd [28].

**Table 2.** Indicators of disease progression and host response in A/Ncr and C57BL/6 mice infected with ECTV via the IN, SC or FP route^1^.

**Marker**	**Route**	**A/Ncr**	**C57BL/6**
**Disease Progression**			
Day of death	IN	7–8 (1000 PFU); 7–12 (20 PFU)	9–14 (1000 PFU);
	FP	7–8 (1000 PFU)	N/A
Weight change	IN	Lose weight from day 5 (5–1000 PFU	Lose weight from day 7 (1000 PFU)
	FP	Lose weight from day 5 (1000 PFU)	N/A
Infectivity titres	IN	Day 2 spleen, liver and lung (1000 PFU)	Day 3 liver; day 4 spleen and lung (1000 PFU)
	FP	Day 2 spleen; day 4 liver; and day 6 lung (1000 PFU)	N/A
ALT/AST	IN	Day 6 (1500 PFU)	>Day 7 (1500 PFU); day 5 (1x10^6^ PFU)
Blood viral DNA	IN	Day 6 (5 PFU)	Day 4 (6500 PFU)
	FP	Day 5 (1000 PFU)	-
			
**Host Response**			
IFN-γ	IN	Day 4 (20 PFU)	Day 4 (1×10^6^ PFU)
	SC	-	Day 2 (1×10^6^PFU)
Neutrophilia	IN	By day 8 (20 PFU)	By day 6 (1×10^6^PFU)
PLN IFN-γ	FP	Day 2 (1000 PFU)	Day 1 (1000 PFU)
PLN Rantes	FP	No change (1000 PFU)	Day 1 (1000 PFU)
PLN IL-9	FP	Day 1 (1000 PFU)	No change (1000 PFU)
PLN gene regulation	FP	22 gene changes from 6–24 hours p.i. (1000 PFU)	80 gene changes from 6–24 hours p.i. (1000 PFU)
Antigen presentation	IN	MLN no presentation up to day 3 (1×10^6^ PFU)	MLN no presentation up to day 3 (1×10^6^ PFU)
	FP	PLN no presentation up to day 3 (1×10^6^ PFU)	PLN presentation from day 1 (1×10^6^ PFU)
Spleen mass	IN	No change	Doubled from day 4–7
	FP	No change	Trebled from day 4–7
CD4 splenic intracellular IFN-γ	FP	2×10^5^ IFN-γ + cells by day 8 (3000 PFU attenuated virus EV-138)	5×10^4^ IFN-γ + cells by day 6 (3000 PFU attenuated virus EV-138)
CD8 splenic intracellular IFN-γ	FP	2.5×10^5^ IFN-γ + cells by day 8 (3000 PFU attenuated virus EV-138)	2.5×10^6^ IFN-γ+ cells by day 6 (3000 PFU attenuated virus EV-138)
Antibody	FP	N/A (1000 PFU)	Seroconversion by day 21

1 Data taken from [29,31,40,42].
